# Norepinephrine acting on adventitial fibroblasts stimulates vascular smooth muscle cell proliferation via promoting small extracellular vesicle release

**DOI:** 10.7150/thno.70974

**Published:** 2022-06-06

**Authors:** Chao Ye, Fen Zheng, Tao Xu, Nan Wu, Ying Tong, Xiao-Qing Xiong, Ye-Bo Zhou, Jue-Jin Wang, Qi Chen, Yue-Hua Li, Guo-Qing Zhu, Ying Han

**Affiliations:** 1 Key Laboratory of Targeted Intervention of Cardiovascular Disease, Collaborative Innovation Center for Cardiovascular Disease Translational Medicine, and Department of Physiology, Nanjing Medical University, Nanjing, Jiangsu 211166, China.; 2 Department of Pathophysiology, Nanjing Medical University, Nanjing, Jiangsu 211166, China.

**Keywords:** norepinephrine, extracellular vesicle, hypertension, adventitial fibroblasts, vascular smooth muscle cell, angiotensin converting enzyme

## Abstract

Excessive sympathetic activity and norepinephrine (NE) release play crucial roles in the pathogeneses of hypertension. Sympathetic fibers innervate adventitia rather than media of arteries. However, the roles of NE in adventitial fibroblasts (AFs) are unknown. This study investigated the roles of NE in regulating AFs-derived extracellular vesicles (EVs) release and vascular smooth muscle cells (VSMCs) proliferation in hypertension.

**Methods:** AFs and VSMCs were prepared from aorta of Wistar-Kyoto rats (WKY) and spontaneously hypertensive rats (SHR). AFs were treated with NE (10 μM) for 24 h (every 6 h, 4 times), and cultured in exosomes-depleted medium for 48 h. EVs were isolated from AFs medium with ultracentrifugation for identification and transfer to VSMCs.

**Results:** NE promoted AFs phenotypic transformation and proliferation, which were prevented by α-receptor antagonist phentolamine rather than β-receptor antagonist propranolol. NE-treated AFs conditioned medium stimulated VSMCs proliferation, which was inhibited by either exosome inhibitor GW4869 or phentolamine. NE increased small EVs number, diameter and angiotensin converting enzyme (ACE) contents. The NE-induced EVs release was abolished by GW4869. The EVs from NE-treated AFs stimulated VSMCs proliferation, which was prevented by angiotensin II type 1 receptor antagonist losartan. The EVs from the ACE knockdown-treated AFs showed lower ACE contents, and lost their roles in stimulating VSMCs proliferation.

**Conclusion:** NE promotes AFs-derived small EVs release and ACE transfer, and then causes VSMCs proliferation in hypertension. Intervention of AFs-derived EVs release may be potential therapeutics for excessive sympathetic activation-related vascular remodeling in hypertension.

## Introduction

Chronic sympathetic over-activity contributes to the pathogeneses of hypertension, and is closely associated with hypertension-related target organ damage and major cardiovascular events 1. Implementation of device-based therapeutic interventions to reduce renal and systemic sympathetic activity is used to attenuate hypertension in patients with resistant hypertension 2. This technique aims to reduce renal sympathetic activation by the destruction of renal sympathetic nerves located in the adventitia of the renal artery 3. Hypertensive patients with arteriolar damage had a higher sympathetic nerve density in the adventitia than those without arteriolar damage 4. Sympathetic activity is directly related to the plasma norepinephrine (NE) level 5 and the severity of the hypertension 6. It is well known that sympathetic nerve terminals release NE. The increase in circulating NE mainly originates from the increase in the sympathetic outflow 7.

Vascular smooth muscle cells (VSMCs) are dominant cells in the media of arteries. VSMCs proliferation greatly contributes to vascular remodeling in hypertension 8-10. Vascular adventitia acts as a biological processing centre for regulating vascular function and structure 11, and may serve as a master regulator of vascular pathophysiology 12. Adventitial fibroblasts (AFs) are the most abundant cell type in the adventitia, which play crucial roles in regulating vascular structure and function 13.

Extracellular vesicles (EVs) are phospholipid membrane surrounded vesicles released by a variety of cells. The EVs play crucial roles in the cell-to-cell communication by transferring protein, mRNA, microRNA and other bioactive molecules in pathophysiological conditions 14-16. EVs regulate cardiovascular function and structure, and are closely associated with the pathogenesis of cardiovascular diseases 17-19. EVs may be a potential tool in the diagnosis, prognosis and therapy of diseases 20-23. We have isolated EVs from rat aortic AFs of Wistar-Kyoto rat (WKY) and spontaneously hypertensive rat (SHR), and showed that AFs-derived EVs play substantial roles in the VSMCs proliferation and migration, and vascular remodeling in hypertension 24-26. In the process of studying AFs-derived EVs, the pattern of sympathetic innervation in arteries aroused our great interest. The sympathetic nerves primarily innervate adventitia of artery, but rarely innervate media of artery 27. The dominant innervation of adventitia are confirmed in primate vertebral artery 28, human arteries in feet 29, internal thoracic artery 30, posterior intercostal artery 31, and renal artery 32. The model of adventitia innervation suggests that NE may play important roles in vascular adventitia. However, the roles of sympathetic activity and NE in the vascular adventitia are almost unknown so far. More interestingly, it is unknown whether NE might may important roles in regulating the vascular AFs-derived EVs release in physiological state and hypertension. The present study is designed to investigate the roles of NE in regulating AFs-derived EVs release impacting on VSMCs proliferation and the underlying mechanisms in physiological state and hypertension.

## Materials and Methods

### Animals

Eight-week-old male WKY and SHR rats were obtained from Vital River Laboratory Animal Technology Co. Ltd (Beijing, China). Experiments were performed in accordance with the Guide for the Care and Use of Laboratory Animals (NIH, 8^th^ edition, 2011), and approved by the Experimental Animal Care and Use Committee of Nanjing Medical University. The criteria for rats used in the present study are that the systolic blood pressure was higher than 150 mm Hg in SHR, and lower than 140 mm Hg in WKY. No animals were excluded after the randomization. The rat was euthanized with pentobarbital sodium (150 mg/kg, iv).

### Culture of AFs and VSMCs

AFs and VSMCs were respectively prepared from thoracic aorta of WKY and SHR. The thoracic aorta was isolated, and a stereomicroscope was used to visualize vascular structure clearly. All vascular branches and perivascular adipose tissues were removed as much as possible. The aorta was cut open longitudinally and the intima was fully scraped off with a sterile blade, and the adventitia was separated from media. Adventitia tissues were treated with 0.2% collagenase-II (Gibco, Carlsbad, CA, USA) in Dulbecco's modified Eagle's medium (DMEM, high glucose; Gibco) for 15-30 min, while the media was digested with 0.4% collagenase-II for at least 60 min. The supernatant was removed after centrifugation and the primary cells were suspended in DMEM with 20% fetal bovine serum (FBS; Gibco), 100 IU/mL penicillin and 10 mg/mL streptomycin in humidified atmosphere containing 5% CO_2_ at 37 °C. The cells were incubated in 10% FBS medium from the second passage. The AFs and VSMCs usually reached the confluency 6 days after seeded into cell culture bottle. The cells were digested with 0.25% pre-warmed trypsin only 1 min for each passage, followed immediately by the addition of a double volume of containing 10% FBS medium to terminate digestion. After centrifugation, the supernatant was discarded, and fresh medium was added again. Then, the cells were mixed and inoculated into a new cell culture bottle for culture. The primary VSMCs and AFs from the 3^rd^ to the 5^th^ passages were used in this study. The purity of AFs and VSMCs used in the present study were over 95%.

### Identification of AFs and VSMCs

Morphology of AFs and VSMCs were examined under light microscopy (Figure S1A-B). And then, the markers of AFs and VSMCs were examined with Western blot. Vimentin, a type III intermediate filament protein, is expressed in mesenchymal cells, which is often used as a marker of fibroblasts 33. α-smooth muscle actin (α-SMA), an isoform of the vascular smooth muscle actins, is typically expressed in the microfilament bundles of VSMCs and serves as a marker of VSMCs 34. Platelet/endothelial cell adhesion molecule-1 (PECAM-1) is highly expressed at endothelial cell-cell junctions and is usually used to distinguish fibroblasts or VSMCs from endothelial cells 35. The VSMCs were identified with positive α-SMA and negative PECAM-1. The AFs were identified with positive vimentin and negative PECAM-1 (Figure S2A-B).

### Isolation of EVs from AFs

EVs were isolated from the medium of AFs using ultracentrifugation method as we previously described 24,26. Briefly, the AFs were seeded into three 75 cm^2^ Cell Culture Flasks (Corning Inc., NY, USA) and the treatments were conducted 24 h when the density of cells reached 65%-70%. After washed with PBS 3 times, the medium was replaced with 25 mL DMEM containing 10% exosomes-depleted FBS (EXO-FBS-50A-1, SBI, Palo Alto, CA, USA) and culture for 48 h. Then, the conditioned medium (CM) was collected and successively centrifuged to remove dead cells (300×g, 5 min), cell debris (3,000×g, 30 min), large vesicles and apoptotic bodies (10,000×g, 60 min). Finally, 70 mL supernatants were injected into a centrifuge tube (Beckman Coulter, Inc., Brea, CA, USA) and ultracentrifuged to pellet the EVs (120,000×g, 70 min, 4 °C) using an OptimaTM L-100 XP Ultracentrifufe (Beckman Coulter). The pellets were washed 3 times with PBS, and resuspended in 200 μL of PBS for molecular assay, or suspended in 200 μL of Radio Immunoprecipitation Assay (RIPA) Lysis Buffer (Thermo-Fisher Scientific, Wilmington, DE, USA) for Western blot assay, or suspended in 1 mL Buffer MZ (Tiangen Biotech, Beijing, China) for total RNA extraction. The EVs were used directly as far as possible, or stored at 4 °C for up to 2 days, or at -80 °C for up to 3 months.

### Determination of EVs amount

In order to compare the EVs amount, cell cultures of each sample were simultaneously carried out as we mentioned above, and 70 mL medium of each sample was collected for the isolation of EVs. The isolated pellet of each sample was suspended in 200 μL solution. Same volume of EVs solution was used for measurements. Three methods were used to evaluate EVs amount as follows: (1) protein content in EVs was used as an index of the amount of EVs 36-38.The pellet was suspended in 200 μL of PBS, and then, 10 μL EVs suspension was mixed with 10 μL RIPA Lysis Buffer for extracting EVs protein. Total protein contents in EVs were detected with Pierce BCA Protein Assay Kit (Thermo Fisher Scientific) following the manufacturer's protocol. The EVs concentration was expressed as μg protein/mL. (2) The amount of EVs-associated protein markers were used to evaluate the EVs amount. The measurement was made with Western blot analyses, and calnexin was used as a negative control. The pellet was suspended in 200 μL RIPA Lysis Buffer, and mixed with 50 μL SDS-PAGE Sample Loading Buffer (5×). Loading amount for each sample was accurately controlled at 30 μL. The relative amount of EVs-associated protein markers (CD9, CD63 and TSG101) represented the amount of EVs. (3) EVs count in the images of transmission electron microscopy (TEM) was used to represent the amount of EVs. The pellet was suspended in 200 μL of PBS. 20 μL of EVs suspension was loaded onto a carbon-coated copper grid (Nisshin EM Corporation, Tokyo, Japan), and fixed about 5 min. Then, the EVs were stained with 2% phosphotungstic acid for 2 min and dried for 10 min. The grids were visualized with transmission electron microscope (Tecnai G2 Spirit TEM, Zeiss, Oberkochen, Germany) at 120 kV. At least 9 TEM images in each group from different batches of EVs isolation were taken for analyses. Photography was taken for one randomly selected field in each electron microscopic section of samples. The number of EVs in images was counted, and expressed as the average number of EVs per μm^2^.

### Measurement of EVs size and distribution

The size of EVs in the TEM images was measured by diameter with ImageJ software (1.8.0, National Institutes of Health: Bethesda, MD, USA). The EVs size was expressed as average diameter in nanometer (nm). Ordinate in EVs size distribution represents the average number per 5 nm as percentage of the total. The data were obtained by analyzing at least 9 TEM images in each group from different batches of EVs isolation.

### Measurement of miR-155-5p and miR-135a-5p levels in EVs with qRT-PCR

EVs pellet was suspended in 1 mL Buffer MZ (Tiangen Biotech) for total RNA extraction using a miRcute miRNA isolation Kit (Tiangen Biotech) following the manufacturer's instructions. Total RNA was quantified by the NanoDrop 2000 Spectrophotometer (Thermo-Fisher Scientific). 1 μg of total RNA of each sample was reverse-transcribed to cDNA by a miRcute Plus miRNA First-Strand cDNA Kit (Tiangen Biotech). Amplification and detection of the PCR products were performed on a StepOnePlus™ Real-Time PCR System (Applied Biosystems, Foster City, CA, USA). U6 small RNA was used as an internal control. The primers were listed in the online supplementary data (Table S1).

### NE treatment and experimental protocols

**Figure 1:**
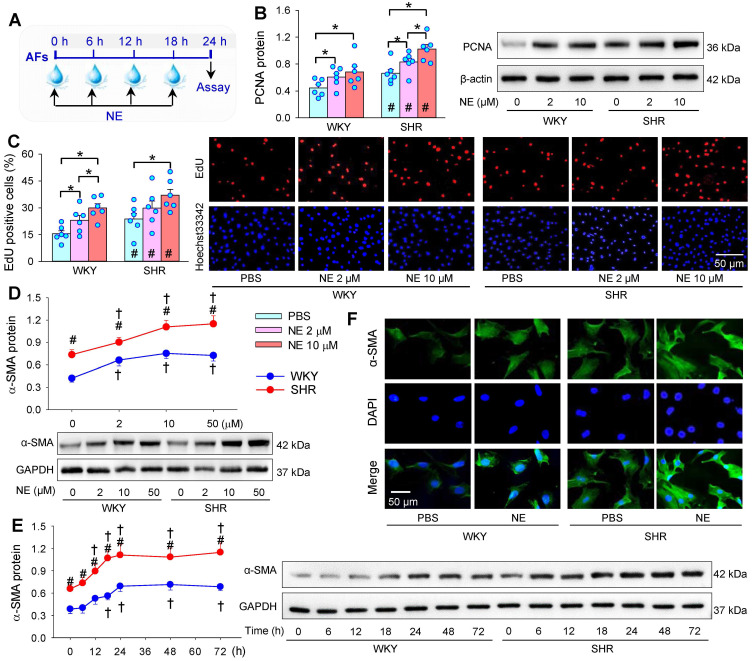
** Effects of norepinephrine (NE) on AFs proliferation and phenotypic transformation of WKY and SHR. (A)** Schematic diagram showing experimental protocols. The AFs were treated with PBS or NE for 24 h. **(B & C)** Effects of different concentration of NE (2 or 10 μM) on AFs proliferation of WKY and SHR. AFs proliferation was evaluated by PCNA protein expression (B) and EdU-positive cells (C). **(D)** Dose-effect of NE (2, 10 and 50 µM) on α-SMA protein expression in AFs of WKY and SHR. **(E)** Time-effect of NE (10 µM) on α-SMA protein expression in AFs of WKY and SHR. **(F)** Immunofluorescence staining for α-SMA (green) in AFs of WKY and SHR showing the AFs phenotypic transformation-induced by the NE treatment (10 μM) for 24 h. The nuclei of cells were stained with DAPI (blue). Data represent the mean ± SEM from 6 independent experiments. *P < 0.05; †P < 0.05 vs 0 μM or 0 h; #P < 0.05 vs WKY; Two-way ANOVA followed by Bonferroni post hoc test.

Norepinephrine bitartrate monohydrate (NE, 10 μM, MedChem Express, Monmouth Junction, NJ, USA) was added into the medium of AFs every 6 h for 4 times. Before each addition of NE, the medium of AFs was gently sucked out with a pipette, and washed with 37 °C PBS for 3 times. Then, fresh medium and NE were immediately added to the AFs for continuous culture (Figure 1A). The concentration of NE at 10 μM was selected for the present study primarily based on the dose-effects of NE (Figure 1B-D), which was consistent with the most of previous studies *in vitro* 39-41. The time-effect of NE on AFs phenotypic transformation showed that the maximum and steady effect occurred at about 24 h after first administration of NE (Figure 1E). However, the half-life of NE is relative short 42. Therefore, NE was added into the medium of AFs every 6 h for 4 times. Such an administration method not only helped to keep an effective concentration of NE for 24 h, but eliminated the excessive metabolites of NE in time to avoid their interference with the experimental results.

### Administration of EVs

Effects of EVs were examined by direct administration of EVs into the VSMCs medium, and the final concentration of EVs in the medium was 30 μg protein/mL. This concentration was selected based on previous studies. Our previous studies have shown that EVs from AFs of SHR dose-dependently promoted VSMCs migration and proliferation, almost reaching its maximal effects at the concentration of 30 µg protein/mL. The final concentration of EVs in the VSMC medium at 30 μg protein/mL medium was about 0.56×10^6^ particles of EVs/mL medium 24-26. The concentration of EVs was similar to other studies, and the protein level of EVs is positive related to the number of EVs 36-38. Moreover, the toxicity of EVs on the VSMCs has been examined by MTT assay. EVs from AFs of WKY or SHR had no significant toxic effects on the VSMCs 26.

### Evaluation of VSMCs proliferation

VSMCs proliferation was evaluated with cell counting kit-8 (CCK8) kit, 5-ethynyl-2'-deoxyuridine (EdU) incorporation assay, and proliferating cell nuclear antigen (PCNA) protein expression as we previously reported 43. For CCK8 assay, cells were seeded into a 96-well plate. CCK8 solution (10 μL) was added into the medium, and cultured in dark at 37 °C for 30 min. A microplate reader (ELX800, BioTek, Vermont, USA) was used for measuring the absorbance at 450 nm. EdU assay was determined by a Cell-Light^TM^ EdU Apollo®567 *In vitro* Imaging Kit (Guangzhou RiboBio, Guangzhou, China). Cells (5×10^3^ ~ 1×10^4^) were cultured in a 96-well plate and 0.1 μL EdU solution was added at 37 °C for 2 h. Then, cells were stained with Apollo and Hoechst-33342 for 30 min, respectively. The images were captured by a fluorescence microscope (Carl Zeiss Meditec, Jena, Germany). The EdU positive cells were counted and normalized by the total number of Hoechst-33342-stained cells.

### Knockdown of angiotensin converting enzyme (ACE) in AFs

Four lentiviral vectors targeting ACE (ACE-siRNA, 1×10^9^ TU/mL) were constructed and verified by Shanghai Genechem (Shanghai, China). A 21-nucleotide (5′-TGCCACGGAGGCCATGATAAA-3′) siRNA targeting ACE was used against the rat ACE mRNA (GenBank accession number NM_012544) in the present study, which down-regulated ACE expression by about 75%. Its validity for ACE knockdown has been identified in our previous study 25. Scrambled siRNA, which was used as negative control in the experiment, is an irrelevant siRNA with random nucleotides. AFs were infected with ACE-siRNA-lentivirus (MOI = 80) containing polybrene for 24 h. The medium was replaced with conventional culture medium for 48 h. The infection efficiency was evaluated by the GFP fluorescence under fluorescence microscope (Axio Vert. Al, Zeiss, Germany). AFs were trypsinized and washed with PBS, and then seeded onto the cell culture bottle and treated with ACE-siRNA plus PBS or NE for 24 h. And then, the media was replaced with 10% exosomes-depleted FBS for 48 h. The culture medium was collected for EVs isolation.

### Immunofluorescence staining

Immunofluorescence staining was used to detect α-SMA expression in the AFs. Cells were plated on glass coverslips in 6-well plates and treated with PBS or NE for 24 h. AFs successively treated with 4% paraformaldehyde for 15 min, 0.2% Triton X-100 for 5 min, and 1% BSA at room temperature for 2 h. Then, cells were incubated at 4 °C with primary antibody against α-SMA (1:200; Protein Tech Group Inc. Chicago, USA) overnight. The cells were then incubated with fluorescence-labeled secondary antibodies (1:1000) for 1 h at room temperature. DAPI (1:1000) was used for nuclear staining. A fluorescence microscope was used for image capture.

### Chemicals and antibodies

Norepinephrine bitartrate monohydrate was obtained from MedChem Express (Monmouth Junction, NJ, USA). Propranolol hydrochloride and losartan were bought from Tocris Bioscience (Bristol, Avon, UK). Phentolamine hydrochloride and GW4869 were purchased from Sigma (St. Louis, MO, USA). GW4869 was dissolved in 1% dimethyl sulfoxide (DMSO). PBS containing 1% DMSO was used as the control. Other chemicals were dissolved in PBS.

Antibodies against α-SMA (1:5000), CD9 (1:1000), TSG101 (1:2000), calnexin (1:2000) and ACE (1:1000) were purchased from Abcam (Cambridge, MA, USA). Antibodies against PCNA (1:5000) and PECAM-1 (1:2000) were obtained from Protein Tech Group Inc. Antibody against CD63 (1:1000) was acquired from Wanleibio. (Shengyang, China). Antibody against vimentin (1:2000) was purchased from Abways Technology, Inc. (Shanghai, China). Antibodies against β-actin (1:10000) and GAPDH (1:10000) were bought from Cell Signaling Technology (Beverly, MA, USA). These antibodies were used for Western blot analyses. GAPDH and β-actin were used as loading control.

### Statistical Analysis

Experiments were performed in a randomized and double-blinded fashion. The number of each group represents the number of samples from different rats. All data are presented as mean ± SEM. One-way ANOVA followed by Bonferroni post hoc test was used for testing the effect of one independent variable or factor. Two-way ANOVA followed by Bonferroni post hoc test was used for examining the effect of two independent variables or factors on a dependent variable. Before ANOVA analyses, the distribution of all data was checked. All data in this study showed normal distribution. Power analysis was used to determine the appropriate sample size required for reliable and reproducible results in the investigating EVs size, number and total protein content in the EVs. P value less than 0.05 was considered as statistical significance.

## Results

### Effects of NE on AFs proliferation and phenotypic transformation

NE was added into AFs medium every 6 h for 4 times. The medium containing NE was replaced with fresh medium and NE each time (Figure 1A). AFs proliferation was evaluated with PCNA protein expression (Figure 1B), EdU incorporation assay (Figure 1C) and CCK8 kit (Figure S3). Enhanced VSMCs proliferation was found in SHR compared with that in WKY. Either 2 μM or 10 μM of NE promotes AFs proliferation of WKY and SHR, and the effect of high concentration of NE was greater than that of low concentration of NE. It is known that phenotypic transformation of vascular AFs to myofibroblasts plays an important role in vascular remodeling 44. α-SMA is used as a marker of the AFs phenotypic transformation 45-47. The α-SMA protein was upregulated in SHR compared with that in WKY. NE promoted α-SMA protein expressions of both WKY and SHR, almost reaching its maximal effect at the concentration of 10 μM (Figure 1D). The NE increased α-SMA protein expression in a time-dependent manner in the NE-treated 24 h. In the following 48 h without NE treatment, the α-SMA protein expression remains at a high level, suggesting that NE-induced phenotypic transformation lasts at least more than 2 days after withdrawal of NE (Figure 1E). The concentration of NE at 2 μM is slightly higher than the plasma NE levels in WKY 48-50. Therefore, the concentration of NE at 10 μM is considered to be optimal and physiologically relevant. This concentration of NE was used in the present study, which was consistent with the most of previous studies *in vitro* 39-41. Immunofluorescence analyses confirmed the NE-induced α-SMA upregulation almost in all AFs of WKY and SHR (Figure 1F).

### Roles of α- and β-receptors in the effects of NE

**Figure 2:**
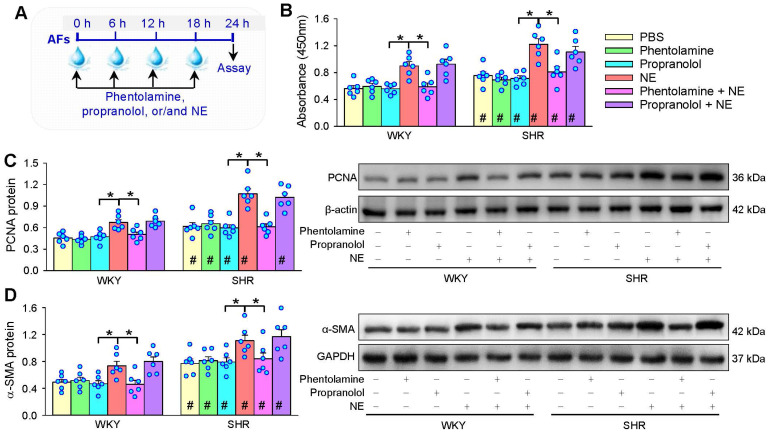
** Roles of α-receptors and β-receptors in NE-induced AFs proliferation and phenotypic transformation of WKY and SHR. (A)** Schematic diagram showing experimental protocols. AFs were incubated with PBS, α-receptor antagonist phentolamine (3 µM), β-receptor antagonist propranolol (3 µM), NE (10 µM), phentolamine plus or propranolol plus NE for 24 h. The proliferation of AFs was evaluated by CCK-8 kits** (B)** and PCNA protein expression **(C)**. AFs phenotypic transformation was determined by α-SMA protein expression **(D)**. Values represent the mean ± SEM from 6 independent experiments. *P < 0.05; #P < 0.05 vs WKY. Two-way ANOVA followed by Bonferroni post hoc test.

α-receptor antagonist phentolamine and β-receptor antagonist propranolol were used to determine the roles of α- and β-receptors in the effects of NE on AFs proliferation and phenotypic transformation (Figure 2A). Either phentolamine or propranolol alone had no significant effects on AFs proliferation and phenotypic transformation. However, the roles of NE in promoting AFs proliferation and phenotypic transformation of WKY and SHR were prevented by phentolamine rather than propranolol (Figure 2B-D). These results indicate that NE promotes AFs proliferation and phenotypic transformation of WKY and SHR mediated by α-receptors.

### Roles of NE-treated AFs conditioned medium on VSMCs proliferation

**Figure 3:**
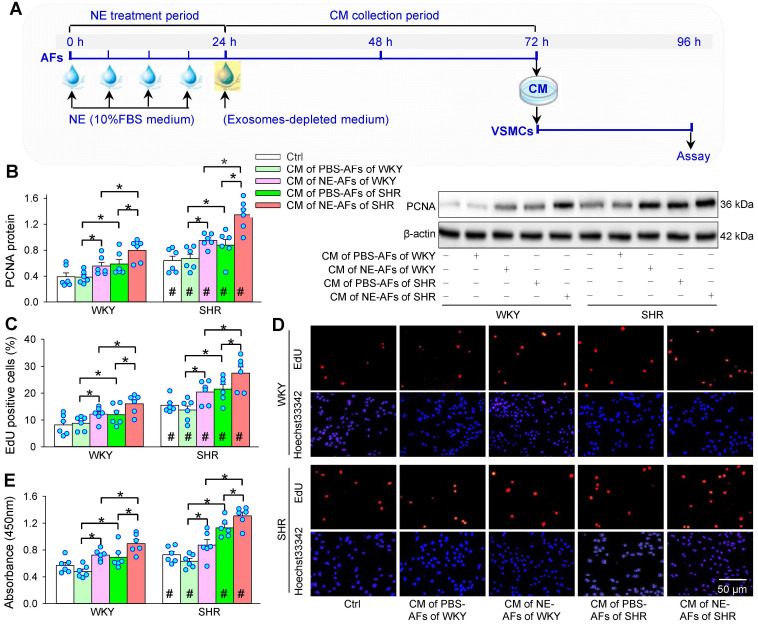
** Effects of conditioned medium (CM) of PBS- or NE-treated AFs from WKY and SHR on VSMCs proliferation of both strains. (A)** Schematic diagram showing experimental protocols. AFs were treated with PBS or NE (10 µM) for 24 h. Media were replaced with exosomes-depleted medium and cultured for 48 h. Then, the medium was transferred to VSMCs for 24 h. **(B)** VSMCs proliferation evaluated by PCNA protein expression. **(C & D)** VSMCs proliferation evaluated by percentage of EdU-positive cells. **(E)** VSMCs proliferation determined with CCK-8. Values represent the mean ± SEM from 6 independent experiments. *P < 0.05; #P < 0.05 vs WKY. Two-way ANOVA followed by Bonferroni post hoc test.

In order to determine whether NE-treated AFs have an impact on VSMCs proliferation, AFs were treated with PBS or NE for 24 h, and the media was replaced with exosomes-depleted medium for 48 h. Then, the conditioned medium (CM) was transferred to VSMCs for 24 h (Figure 3A). The CM of AFs from SHR promoted VSMCs proliferation of both WKY and SHR, while the CM of AFs from WKY had no significant effects on VSMCs proliferation. Importantly, the CM of NE-treated AFs of WKY and SHR promoted VSMCs proliferation of WKY and SHR (Figure 3B-D).

### Effects of GW4869 on NE-induced VSMCs proliferation

**Figure 4:**
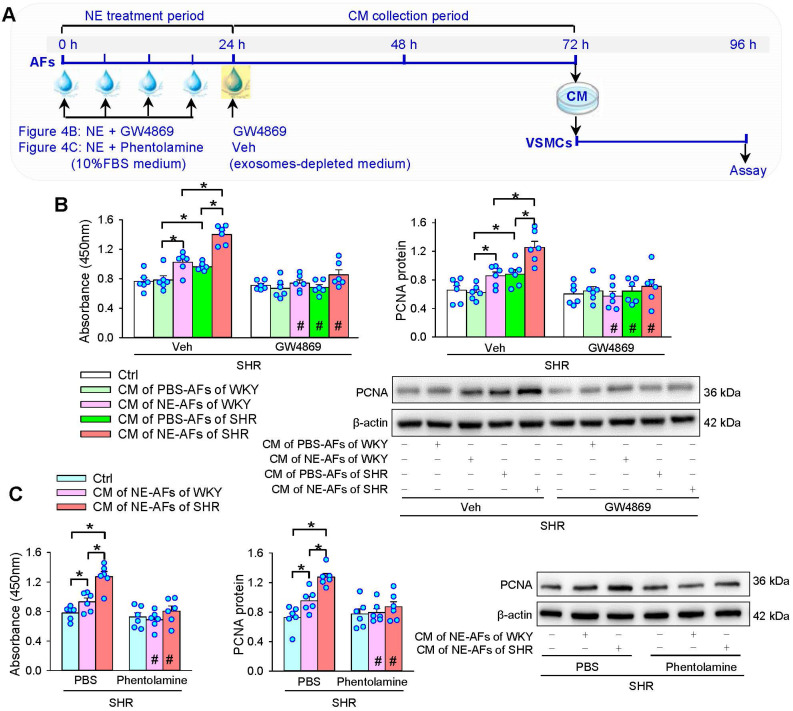
** Effects of exosome inhibitor GW4869 and α-receptor antagonist phentolamine on the roles of the CM of NE-treated AFs from WKY and SHR in regulating VSMCs proliferation of SHR. (A)** Schematic diagram showing experimental protocols. AFs were treated with PBS or NE (10 µM) for 24 h. GW4869 (20 µM) was added to exosomes-depleted medium and cultured for 48 h. Then, the medium was transferred to VSMCs for 24 h. **(B)** Effects of GW4869 on the roles of the CM of NE-treated AFs from WKY and SHR in regulating VSMCs proliferation of SHR. **(C)** Effects of phentolamine on the roles of the CM of NE-treated AFs from WKY and SHR in regulating VSMCs proliferation of SHR. Phentolamine (3 µM) was added immediately before each NE administration. Values represent the mean ± SEM from 6 independent experiments. *P < 0.05; #P < 0.05 vs Veh or PBS. Two-way ANOVA followed by Bonferroni post hoc test.

To determine whether EVs are involved in the roles of CM from NE-treated AFs of WKY and SHR in promoting VSMCs proliferation, an exosome inhibitor GW4869 was added into the CM along with NE 4 times in 24 h to inhibit the production of EVs. And then, the media was replaced with exosomes-depleted medium containing GW4869 for 48 h (Figure 4A). GW4869 prevented not only the roles of CM of AFs of SHR, but also the roles of CM from NE-treated AFs of WKY and SHR in promoting VSMCs proliferation of SHR (Figure 4B). Moreover, α-receptor antagonist phentolamine was used to identify whether α-receptors mediate the effects of the NE on the CM of AFs. Phentolamine abolished the roles of the CM from NE-treated AFs of WKY and SHR on the VSMCs proliferation of SHR (Figure 4C). These results suggest that NE-induced EVs release from AFs may contribute to VSMCs proliferation of WKY and SHR via activating α-receptors in AFs.

### Effects of NE on the EVs release from AFs

**Figure 5:**
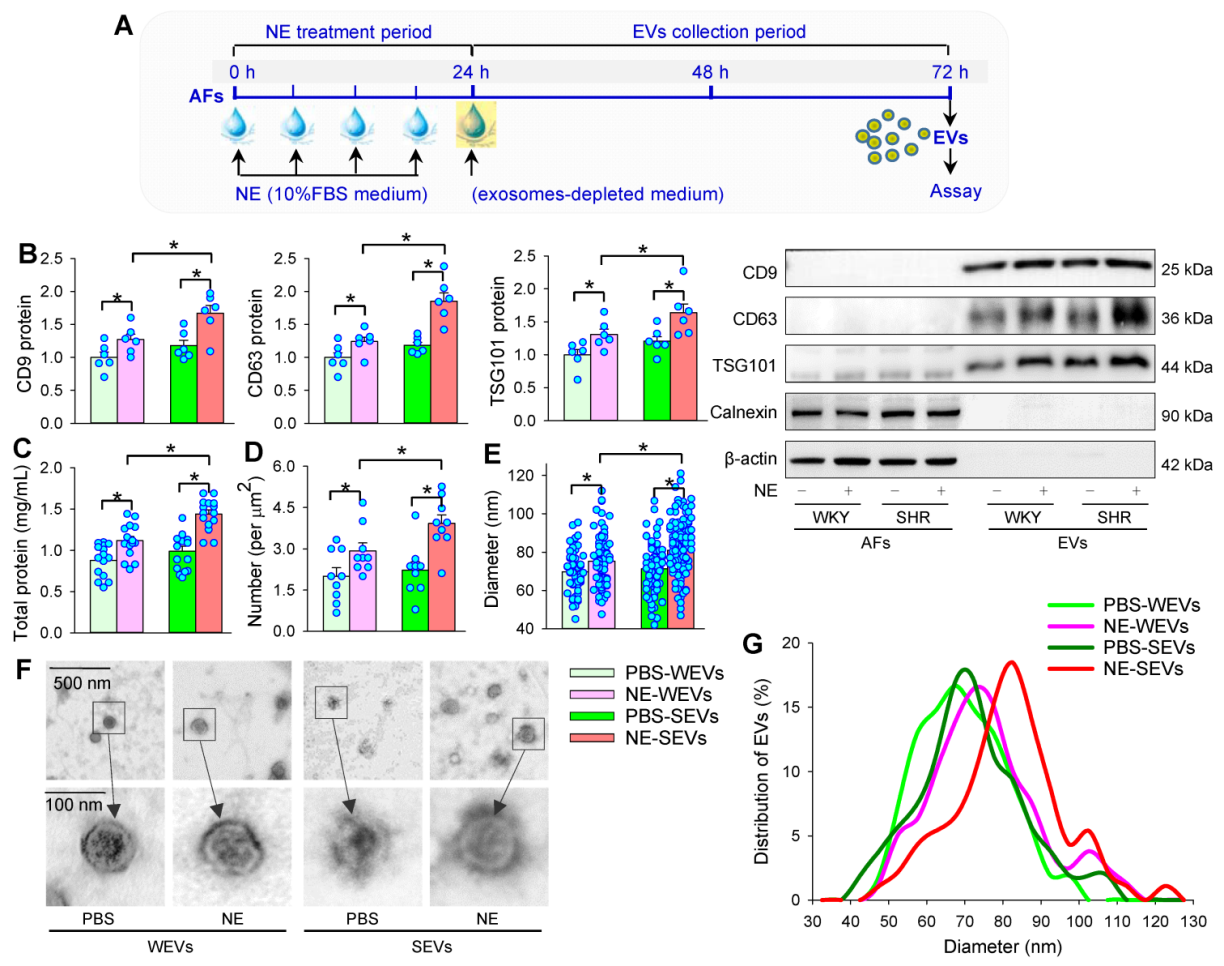
** Effects of NE on EVs release from AFs in WKY and SHR. (A)** Schematic diagram showing experimental protocols. AFs were treated with PBS or NE (10 µM) for 24 h, and then, the media were replaced with exosomes-depleted medium and cultured for 48 h for isolating EVs. **(B)** EVs biomarkers (CD9, CD63 and TSG101) measured with Western blot analyses. Calnexin and β-actin were used as negative controls. The value was normalized by the protein level of PBS-WEVs. n = 6 per group. **(C)** Total protein content in EVs. n = 15 batches of isolation per group. **(D)** Mean number of EVs obtained from the analyses of 36 transmission electron microscopic (TEM) images. n = 9 per group. **(E)** Mean diameter of EVs obtained from the analyses of 36 TEM images. n = 54, 79, 73 and 134 respectively. **(F)** Representative TEM images of WEVs and SEVs. **(G)** Distribution of different size of WEVs and SEVs according to the analyses of 36 TEM images. n = 9 per group. Values represent the mean ± SEM. *P < 0.05. Two-way ANOVA followed by Bonferroni post hoc test. WEVs, EVs from WKY; SEVs, EVs from SHR.

**Figure 6:**
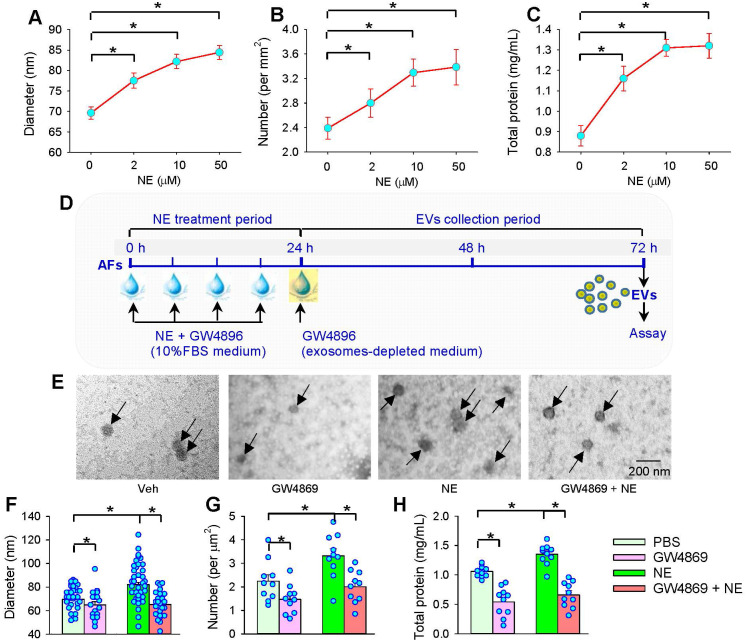
** Dose-effects of NE on EVs release and effects of exosome inhibitor GW4869 on NE-induced EVs release from AFs of SHR. (A-C)** Dose-effects of NE (0, 2, 10 and 50 µM) on EVs release from AFs of SHR. (A) Mean diameter of EVs obtained from the analyses of 18 transmission electron microscopic (TEM) images for each group. (**B**) Mean number of EVs obtained from the analyses of 18 TEM images for each group. (**C**) Total protein content in EVs of SHR obtained from 10 batches of isolation per group. **(D)** Schematic diagram showing the experimental protocols for the effects of GW4869 on EVs release. AFs were treated with vehicle (Veh), GW4869 (20 μM), NE (10 µM) or NE+GW4869 for 24 h, and then, the media was replaced with exosomes-depleted medium and cultured for 48 h for isolating EVs. PBS containing 1% DMSO was used as the control (Veh). **(E-H)** Effects of exosome inhibitor GW4869 on NE-induced EVs release from AFs of SHR. (**E**) Representative TEM images of EVs from AFs of SHR. The arrows indicate EVs. (**F**) Mean diameter of EVs obtained from the analyses of 10 TEM images for each group. (**G**) Mean number of EVs obtained from the analyses of 10 TEM images for each group. (**H**) Total protein content in EVs of SHR obtained from 10 batches of isolation per group. Values represent the mean ± SEM. *P < 0.05. One-way ANOVA followed by Bonferroni post hoc test.

AFs were treated with NE for 24 h. After the AFs were washed 3 times, the medium was replaced with exosomes-depleted medium and cultured for 48 h for the isolation of EVs to determine the effects of NE on EVs release (Figure 5A). The isolated EVs were confirmed by three positive EVs-associated protein markers (CD9, CD63 and TSG101), and a negative endoplasmic reticulum marker calnexin 24. To determine the effects of NE on the EVs release, same volume of EVs solution were measured with 3 different methods including EVs-associated protein expressions, total protein concentration of EVs, and TEM-based EVs number and diameter measurement in the EVs from AFs of WKY (WEVs) and the EVs from AFs of SHR (SEVs). According to Power analysis, we could detect significant change at a power of 0.95 with a total sample size of more than 24 TEM images (6 for each group), 28 TEM images (7 for each group) and 28 batches of isolation of EVs (7 for each group) for determining the difference of EVs size and diameter, and total protein concentration. Therefore, the sample size used for measuring EVs number, size and total protein was bigger than the predicted value of Power analysis. NE increased CD9, CD63 and TSG101 protein levels (Figure 5B), total protein concentration (Figure 5C), and the number of EVs in TEM images (Figure 5D) of both WKY and SHR. Furthermore, NE increased the diameter of WEVs (75.33 ± 1.58 nm vs. 69.72 ± 1.18 nm, *P* < 0.05) and SEVs (80.98 *±* 1.44 nm vs. 71.27 *±* 1.92 nm, *P <* 0.05) compared with PBS treatment (Figure 5E). The TEM images showed that the EVs from NE-treated AFs of WKY and SHR had a similar spherical or cup-shaped appearance to the EVs from PBS-treated AFs (Figure 5F). The distribution curve of different size of SEVs was similar to WEVs. However, NE treatment caused a right shift of the distribution curve of both WEVs and SEVs. According to the classification of EVs subtypes in the guidelines of MISEV2018 51 and the size distribution curve, the vast majority of EVs derived from AFs in the present study belong to small EVs (Figure 5G). Although there was no significant difference in the EVs number, diameter, total protein contents and EVs marker levels between the PBS-treated WEVs and SEVs, these values were significantly higher in the NE-treated SEVs than those in the NE-treated WEVs (Figure 5B-G). We further examined the dose-effects of NE on the EVs release from the AFs of SHR. NE increased the EVs diameter, number and total protein content in the EVs, almost reaching its maximal effects at the concentration of 10 μM of NE (Figure 6A-C). These results indicate that NE promotes EVs release in increasing EVs number and size in the AFs of both WKY and SHR.

### Effects of GW4869 on NE-induced EVs release

To confirm the roles of NE in promoting the EVs release from AFs, we further examined whether an exosome inhibitor GW4869 would inhibit the NE-induced EVs release in the AFs of SHR (Figure 6D). As we expected, GW4869 not only reduced the EVs diameter, number and total protein content in the EVs from the AFs of SHR, but reversed the effects of NE in promoting EVs release (Figure 6E-H).

### Effects of EVs from NE-treated AFs on VSMCs proliferation

**Figure 7:**
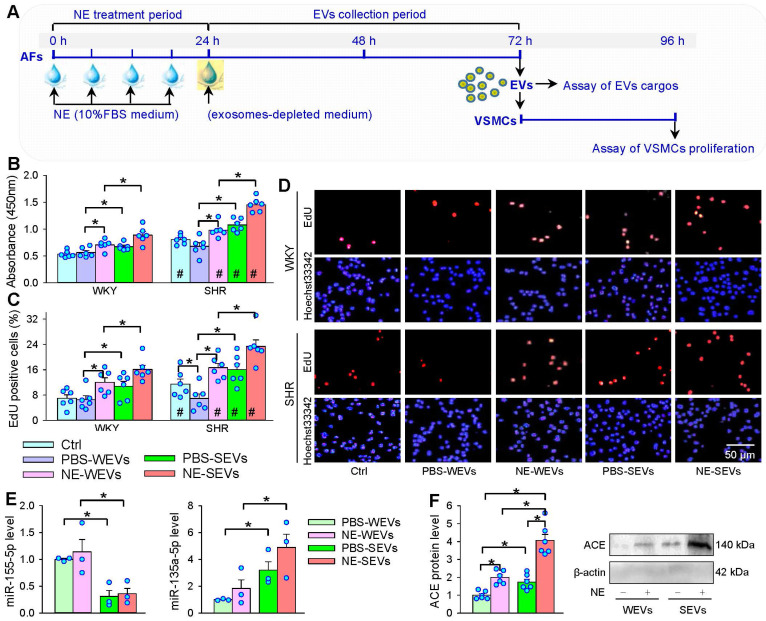
** Effects of EVs from NE-treated AFs on VSMCs proliferation and cargos of EVs. (A)** Schematic diagram showing experimental protocols. AFs were treated with PBS or NE (10 µM) for 24 h. The media was replaced with exosomes-depleted medium and cultured for 48 h for isolating EVs, and then, the EVs (30 µg protein/mL) were transferred to VSMCs for 24 h. **(B-D)** The cell proliferation was evaluated by CCK-8 kits and EdU assay. **(E)** MiR-155-5p and miR-135a-5p levels in WEVs and SEVs. **(F)** ACE protein levels in WEVs and SEVs. The values were normalized by the values of PBS-WEVs. Values represent the mean ± SEM from 3 or 6 independent experiments.*P < 0.05; #P < 0.05 vs WKY. Two-way ANOVA followed by Bonferroni post hoc test. WEVs, EVs from AFs of WKY; SEVs, EVs from AFs of SHR.

In order to determine whether NE has an impact on the roles of EVs in regulating VSMCs proliferation, the effects of EVs from NE-treated AFs on VSMCs proliferation were examined. The EVs were respectively isolated from PBS- or NE-treated AFs of WKY and SHR, and the same concentration of the isolated EVs were added into the VSMCs medium of WKY and SHR (Figure 7A). NE-treated EVs stimulated VSMCs proliferation of WKY and SHR. Although the PBS-treated SEVs promoted VSMCs proliferation, NE further enhanced the role of SEVs in promoting the VSMCs proliferation (Figure 7B-D). These findings indicate that NE-induced EVs release promotes VSMCs proliferation of WKY and SHR. It is interesting to know whether higher number of EVs in NE-treated condition was caused by the higher number of cells in treated condition or by the higher number of EVs released from each cells. We counted the mean number of AFs treated with PBS or NE immediately after collecting the medium for isolating EVs. Compared with PBS treatment, NE increased AFs number and EVs number by 17.4% and 46.3% respectively in WKY, and increased AFs number and EVs number by 25.6% and 76.3% respectively in SHR. The results suggest that the role of NE in promoting EVs release primarily attributes to more EVs release from per cells. NE-induced AFs proliferation may partially contribute to the upregulation of EVs releases.

### Effects of NE on the EVs cargo in regulating VSMCs proliferation

Our previous studies have shown that ACE and miR-135a-5p contents are increased, while miR-155-5p contents are decreased in the SEVs compared with WEVs, which contributes to the roles of SEVs in promoting VSMCs proliferation 24-26. It is interesting to know whether NE affects these signal molecule contents in the SEVs, and which is responsible for the VSMCs proliferation effect. NE had no significant effect on the miR-155-5p content in SEVs, while NE induced a tendency in increasing miR-135a-5p content in SEVs, but the difference did not reach a significant level (Figure 7E). However, NE increased ACE content in the WEVs and SEVs, and the ACE content in the NE-treated SEVs was much more than that in the NE-treated WEVs (Figure 7F).

### Losartan in VSMCs abolishes the effects of EVs from NE-treated AFs

**Figure 8:**
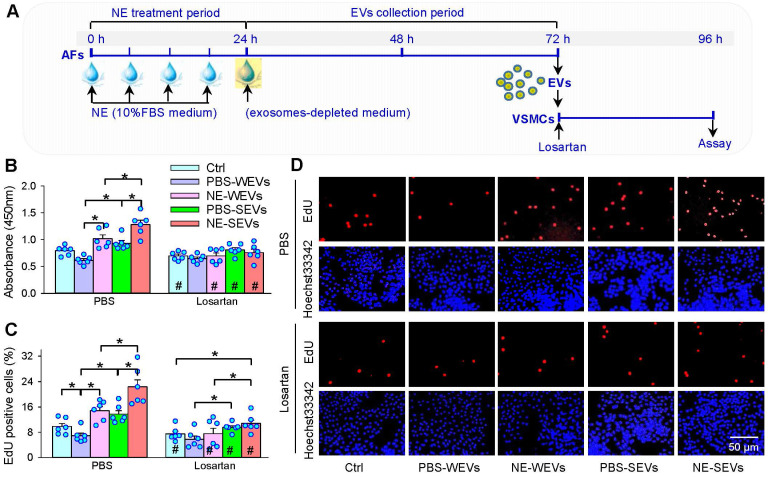
** Effects of losartan on the roles of EVs in VSMCs proliferation of SHR. (A)** Schematic diagram showing experimental protocols. AFs were treated with PBS or NE (10 µM) for 24 h. The media was replaced with exosomes-depleted medium and cultured for 48 h for isolating EVs. VSMCs were pretreated with losartan (10 µM) for 10 min, and then, the EVs (30 µg protein/mL) were transferred to VSMCs for 24 h. **(B)** VSMCs proliferation was evaluated by CCK-8 kits. **(C & D)** VSMCs proliferation was evaluated by EdU assay. Values represent the mean ± SEM from 6 independent experiments. *P < 0.05; #P < 0.05 vs PBS. Two-way ANOVA followed by Bonferroni post hoc test. WEVs, EVs from AFs of WKY; SEVs, EVs from AFs of SHR.

It is well known that ACE promotes angiotensin II (Ang II) production, and the increased Ang II stimulates VSMCs proliferation via activating Ang II type 1 (AT_1_) receptors. Therefore, AT_1_ receptor antagonist losartan was used in VSMCs to determine whether the increased ACE content in the EVs contributes to the roles of NE in the EVs-mediated VSMCs proliferation (Figure 8A). Losartan not only attenuated the roles of SEVs, but also the roles in NE-induced proliferation-promoting effects of WEVs and SEVs (Figure 8B-D). These results suggest that the EVs from NE-treated AFs of WKY and SHR promote VSMCs proliferation via the increased ACE transfer in the EVs. Application of phentolamine in the VSMCs of SHR had no significant effects on the roles of EVs from NE-treated AFs of WKY and SHR in promoting VSMCs proliferation of SHR (Figure S4), excluding the possibility that the effects of WEVs and SEVs may be caused by the residual trace NE.

### ACE knockdown in AFs abolishes the effects of EVs from NE-treated AFs

**Figure 9:**
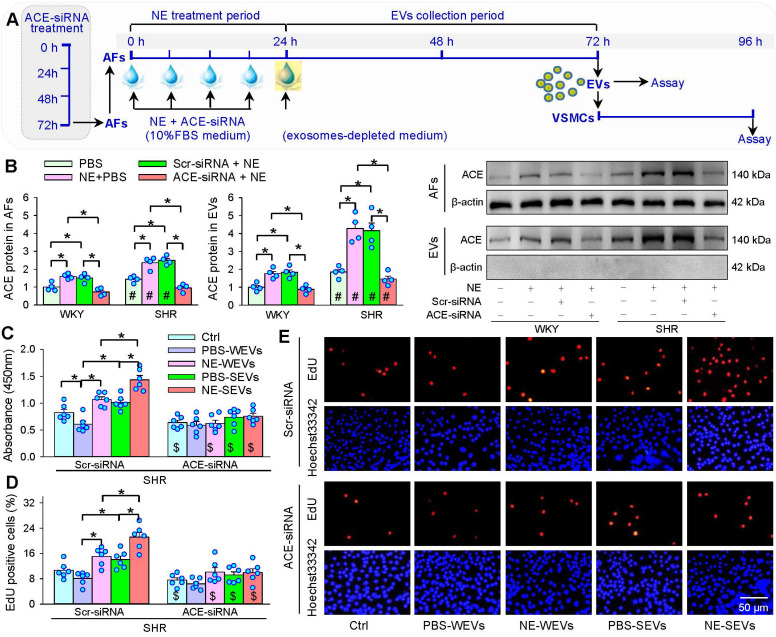
** Effects of ACE knockdown on the roles of EVs in VSMCs proliferation of SHR. (A)** Schematic diagram showing experimental protocols. AFs were infected with control siRNA lentivirus (Scr-siRNA) or ACE-siRNA-lentivirus (ACE-siRNA, MOI = 80) for 72 h. AFs were trypsinized and seeded onto the cell culture bottle and treated with NE (10 µM) plus ACE-siRNA (MOI = 80) or corresponding control for 24 h, and then, the media was replaced with exosomes-depleted medium and cultured for 48 h for isolating EVs. The VSMCs of SHR were treated with EVs (30 µg protein/mL) for 24 h. **(B)** ACE content in AFs and EVs. The measurements were carried out after AFs infected with ACE-siRNA 72 h. **(C-E)** Effects of ACE knockdown with siRNA in AFs on the roles of AFs-derived EVs in regulating VSMCs proliferation in VSMCs of SHR. The cell proliferation was evaluated by CCK-8 kits and EdU assay. Values represent the mean ± SEM from 4 or 6 independent experiments. *P < 0.05; #P < 0.05 vs WKY; $P < 0.05 vs Scr-siRNA. Two-way ANOVA followed by Bonferroni post hoc test. WEVs, EVs from AFs of WKY; SEVs, EVs from AFs of SHR.

ACE knockdown was carried out to further confirm that EVs from NE-treated AFs promote VSMCs proliferation by increased ACE transfer in the EVs (Figure 9A). The ACE knockdown reduced ACE protein levels in both AFs and EVs of both WKY and SHR, indicating the efficiency of ACE knockdown (Figure 9B). The EVs from ACE knockdown-treated AFs of WKY and SHR lost the roles of NE in promoting VSMCs proliferation (Figure 9C-E). These results provide solid evidence that increased EVs-mediated ACE transfer in WKY and SHR mediates the roles of NE in promoting VSMCs proliferation.

## Discussion

AFs in vascular adventitia plays important roles in regulating vascular structure and function 11. Excessive sympathetic activity is closely associated with the pathogeneses of hypertension and related target organ damage 1,52. Sympathetic nerves predominantly innervate vascular adventitia rather than vascular media 27. However, the roles of NE, a vital regulator in neurohumoral regulation, in the vascular adventitia are still unknown. This study first showed the important role of NE in promoting EVs release and regulating angiotensin system activity. The primary novel findings in the present study are that NE promotes EVs release from AFs via activating α-receptors. The NE increases the EVs size and number, and ACE content in the EVs. The NE-induced ACE increase in the EVs contributes to the roles of NE in promoting VSMCs proliferation of WKY and SHR. It is known that sympathetic nerves primarily innervate adventitia of artery, but rarely innervate media of artery 27. The sympathetic nerve activity and plasma NE level were increased in hypertension 1. The persistent sympathetic over-activity in hypertension promotes the NE release to adventitia of artery, and thus increases EVs release from AFs and the ACE contents in the EVs. The increased EVs with more ACE content act on adjacent VSMCs, causing VSMCs proliferation, and thereby contributing to vascular remodeling and hypertension. EVs might be a crucial target for the intervention of sympathetic over-activity-related vascular remodeling in hypertension.

NE was added into the AFs 4 times in 24 h to mimic the persistent sympathetic over-activation, and the repeated administration assured the effective concentration of NE in the AFs medium to avoid the shortage of short half-life of NE. NE-induced AFs phenotypic transformation occurred at least 6 h later, suggesting that NE and sympathetic activity in rapid regulation of vascular contraction and blood pressure is not involved in AFs phenotypic transformation. The AFs phenotypic transformation induced by 24 h-NE treatment lasted at least 48 h after the withdrawal of NE. The findings suggest that the NE-induced AFs phenotypic transformation and proliferation contribute to the roles of NE in promoting EVs release.

The study reveals an important role of NE in the control of EVs release from AFs. The NE-induced EVs release includes the increased size and number of EVs, and the ACE content in the EVs. In measuring the ACE content in the EVs, the same loading amount of EVs samples were determined by the same total protein content of the EVs. NE-induced change of ACE content in the EVs of SHR was much greater than that in AFs of WKY. These findings suggests that NE promotes the AFs packaging ACE in the EVs, and the NE-induced increases in the EVs size and number further augment the amount of ACE transferred by the EVs. Importantly, AT_1_ receptor antagonist losartan in the VSMCs almost abolished the roles of EVs from NE-treated AFs in stimulating VSMCs proliferation. The EVs from ACE knockdown-treated AFs lost their roles in promoting VSMCs proliferation. These results provide solid evidence that NE-induced EVs release and increased ACE transfer in the EVs contributes to VSMCs proliferation. On the other hand, ACE presents as a membrane-bound form in cells or a soluble form in blood and numerous body fluids 53. ACE is known to convert Ang I to Ang II, which contributes to VSMCs proliferation and vascular remodeling in hypertension. Inhibition of ACE reduces VSMCs proliferation in rats 54. It is certain that NE-induced EVs release promotes VSMCs proliferation via transferring ACE to VSMCs. We think that the EVs containing higher ACE content enter the VSMCs and increase the ACE level of VSMCs in both membrane-bound form and soluble form. The up-regulated ACE promotes Ang II production, which in turn act on AT_1_ receptors, causing VSMCs proliferation. Our recent studies have shown that miR-155-5p level in the EVs of SHR is greatly reduced, and miR-155-5p inhibits ACE expression and VSMCs proliferation migration and oxidative stress of SHR 24,55. Fibronectin type III domain containing 5 (FNDC5) is known to attenuate insulin resistance, and improves glucose and lipid metabolism 56-58, while increased miR-135a-5p in the EVs of SHR promotes VSMCs proliferation via inhibiting FNDC5 expression in WKY and SHR 26,59. However, NE failed to induce significant changes in the miR-155-5p and miR-135a-5p levels in the EVs of WKY and SHR, suggesting that miR-155-5p and miR-135a-5p in the EVs are not involved in the roles of NE in promoting VSMCs proliferation. It is noteworthy that the AFs and VSMCs were obtained from aorta of WKY and SHR in the present study. The results may be not necessarily applicable to all VSMCs from other arteries. A limitation in the present study is that the results were only obtained from *in vitro* studies.

**Figure 10:**
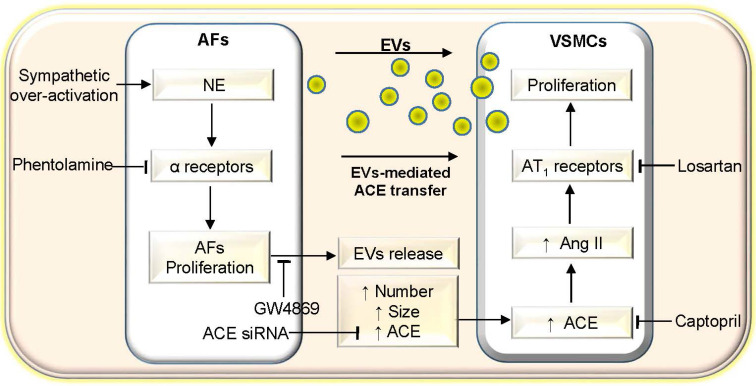
** Schematic diagram showing the roles of NE in AFs-derived EVs in promoting VSMCs proliferation of WKY and SHR.** AFs, adventitial fibroblasts; EVs, extracellular vesicles; VSMCs, vascular smooth muscle cells; NE, norepinephrine; ACE, angiotensin converting enzyme; Ang II, angiotensin II.

In conclusion, NE promotes AFs-derived small EVs release including increasing the EVs size, number and ACE content of WKY and SHR. The EVs from NE-treated AFs of SHR promotes VSMCs proliferation of WKY and SHR via increased ACE transfer in the EVs (Figure 10). The study reveals a novel role and mechanism of NE in AFs-derived small EVs in vascular regulation in physiological state and hypertension. The findings may shed a light on the mechanism of excessive sympathetic activity in vascular remodeling in hypertension. Inhibition of AFs-derived EVs release and ACE transfer in the EVs may be potential therapeutics for excessive sympathetic activation-related vascular remodeling.

## Supplementary Material

Supplementary figures and table.

## Abbreviations

α-SMA: α-smooth muscle actin
ACE: angiotensin converting enzyme
AFs: adventitial fibroblasts
CCK8: cell counting kit-8
CM: conditioned medium
EdU: 5-ethynyl-2'-deoxyuridine
EVs: extracellular vesicles
FBS: fetal bovine serum
NE: norepinephrine
PCNA: proliferating cell nuclear antigen
SHR: spontaneously hypertensive rat
SEVs: EVs from AFs of SHR
TEM: transmission electron microscopy
VSMCs: vascular smooth muscle cells
WKY: Wistar-Kyoto rat
WEVs: EVs from AFs of WKY
