# Supplementary material for: Norepinephrine acting on adventitial fibroblasts stimulates vascular smooth muscle cell proliferation via promoting small extracellular vesicle release
Source: Theranostics. 2022 Jun 6;12(10):4718–33. doi: 10.7150/thno.70974 (PMC9254254; doi:10.7150/thno.70974)
Supplement: Supplementary file 1 — Supplementary figures and table. [file thnov12p4718s1.pdf]

Online Data Supplement

(Figure S1-S4 & Table S1)

Norepinephrine acting on adventitial fibroblasts stimulates vascular smooth muscle cell proliferation via promoting extracellular vesicle release

Chao Ye[1]; Fen Zheng[1]; Tao Xu[1]; Nan Wu[1]; Ying Tong[1]; Xiao-Qing Xiong[1]; Ye-Bo Zhou[1]; Jue-Jin Wang[1]; Qi Chen[2]; Yue-Hua Li[2]; Guo-Qing Zhu [1]*; Ying Han[1]*

[1] Key Laboratory of Targeted Intervention of Cardiovascular Disease, Collaborative Innovation Center for Cardiovascular Disease Translational Medicine, and Department of Physiology, Nanjing Medical University, Nanjing, Jiangsu 211166, China;

[2] Department of Pathophysiology, Nanjing Medical University, Nanjing, Jiangsu 211166, China

Supplemental Figures

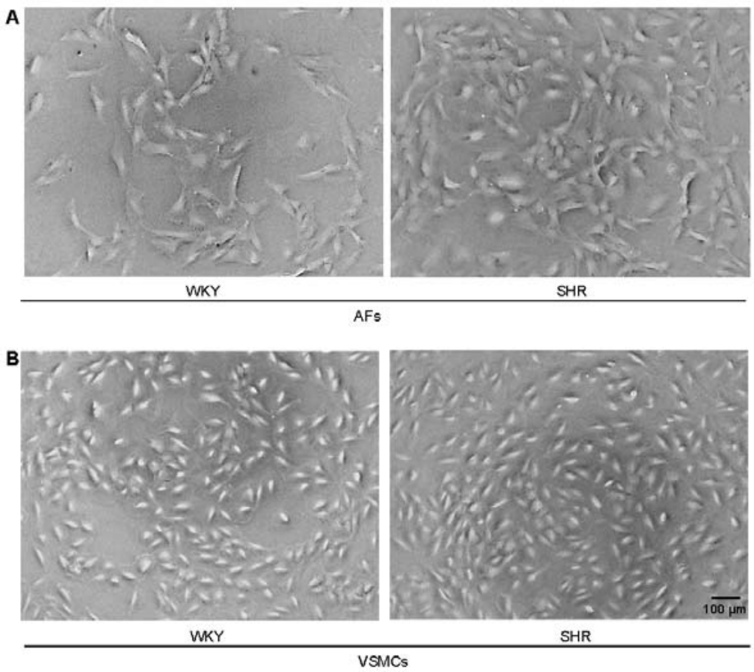

Figure S1. AFs and VSMCs images under light microscopy. (A) AFs of WKY and SHR. (B) VSMCs of WKY and SHR.

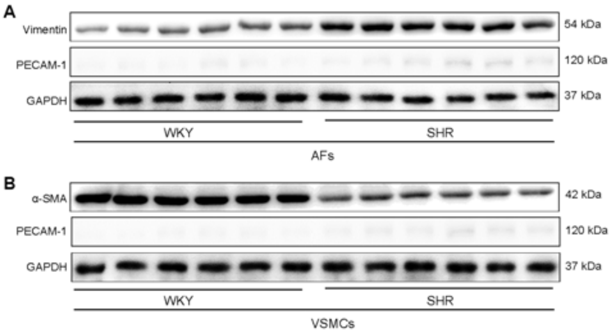

Figure S2 Western blot showing Vimentin, α-SMA and PECAM-1 protein expressions of AFs (A) and VSMCs (B).

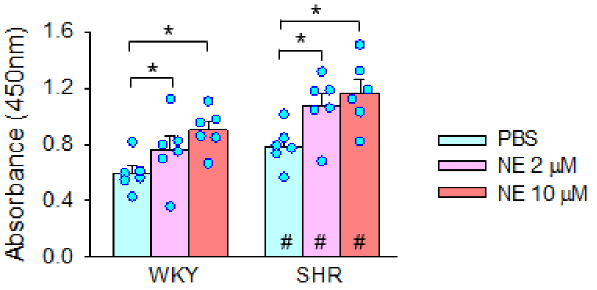

Figure S3. Roles of norepinephrine (NE) in AFs proliferation. The AFs were treated with PBS or NE (2 µM or 10 µM) for 24 h. AFs proliferation was evaluated by CCK-8. Data represent the mean ± SEM from 6 independent experiments. *P < 0.05; #P < 0.05 vs WKY. Two-way ANOVA followed by Bonferroni post hoc test.

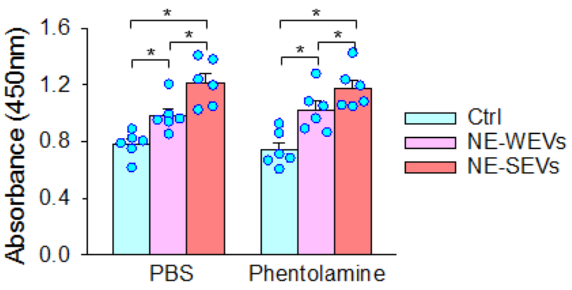

Figure S4. Phentolamine treatment (3 µM) in the VSMCs had no significant effects on the roles of NE-WEVs and NE-SEVs in promoting VSMCs proliferation of SHR. Data represent the mean ± SEM from 6 independent experiments. *P < 0.05. Two-way ANOVA followed by Bonferroni post hoc test. NE-WEVs, EVs from NE-treated AFs of WKY; NE-SEVs, EVs from NE-treated AFs of SHR.

Supplementary Table

Table S1 Primers for qRT-PCR analysis in rats

Name	Primer	Sequence
Rno-miR-135a-5p		5’-GTGGTTTGTCCAAACTCATC-3’
Rno-miR-155-5p		5’-CGTTAATGCTAATTGTGATAGGGGT-3’
Rno-miR-U6		5’- TTGGAACGATACAGAGAAGATTAGCAT-3’
